# Development and validation of a new nomogram model for predicting acute ischemic stroke in patients with non-valvular atrial fibrillation

**DOI:** 10.3389/fnins.2026.1626825

**Published:** 2026-03-19

**Authors:** Shiyu Jiang, Danni Chen, Qiong Wu, Chao Jiang, Yukun Ping, Jie Zhao, Linlin Xie, Xiaobo Li

**Affiliations:** 1 Department of Neurology, Northern Jiangsu People’s Hospital Affiliated to Yangzhou University, Yangzhou, China; 2 Department of Graduate School, Xuzhou Medical University, Jiangsu, China; 3 Medical College of Yangzhou University, Jiangsu, China

**Keywords:** acute ischemic stroke, CHA_2_DS_2_-VASc score, clinical risk prediction model, nomogram, non-valvular atrial fibrillation

## Abstract

**Background and objectives:**

Non-valvular atrial fibrillation (NVAF) significantly increases the risk of acute ischemic stroke (AIS). Current risk prediction models have limitations in comprehensively capturing the multidimensional factors contributing to stroke risk. This study aimed to establish a novel nomogram model for predicting AIS in NVAF patients by integrating comprehensive parameters including clinical characteristics, cardiac anatomical features, functional indices, electrophysiological patterns, hemodynamic parameters, and serum biomarkers.

**Methods:**

We conducted a retrospective study of 415 NVAF patients from Northern Jiangsu People’s Hospital. After applying inclusion and exclusion criteria, 374 patients (193 with AIS) were randomized into 7:3 training/testing cohorts. Variables with *P* < 0.2 in univariate analysis were entered into LASSO regression, followed by review and validation by three senior clinical experts in neurology and cardiology, and then subjected to multivariate logistic regression to identify independent risk factors for nomogram construction. Model performance was comprehensively evaluated through receiver operating characteristic (ROC) curve analysis, calibration curves, and decision curve analysis (DCA) with bootstrap resampling (1,000 iterations). The predictive performance of the new nomogram model was compared with the CHA_2_DS_2_-VASc score using net reclassification improvement (NRI), integrated discrimination improvement (IDI), ROC analysis, calibration curves, and decision curves.

**Results:**

Eight variables were identified as independent predictors of AIS in NVAF patients: age, admission systolic blood pressure (SBP), history of stroke, anticoagulant therapy, left atrial diameter (LAD), left atrial appendage (LAA) filling defect, white blood cell count (WBC), and D-dimer levels (all *P* < 0.05). The nomogram incorporating these parameters demonstrated excellent discrimination (AUC: 0.852 in training cohort, 0.847 in testing cohort), calibration, and clinical utility. Compared to the CHA_2_DS_2_-VASc score, the new model showed superior predictive performance across all evaluation metrics.

**Conclusion:**

The developed nomogram model, which integrates clinical, anatomical, functional, and laboratory parameters, demonstrates superior prediction performance compared to the conventional CHA_2_DS_2_-VASc score for AIS risk stratification in NVAF patients. This multidimensional approach may facilitate more personalized and precise risk assessment to guide preventive strategies.

## Introduction

Atrial fibrillation (AF) serves as a primary risk factor for cardioembolic stroke (Ozdemir et al., 2023), conferring a 3–5-fold increase in ischemic stroke risk (Maida et al., 2020). Furthermore, AF-associated AIS exhibits higher recurrence rates, greater disability burdens, and elevated mortality compared to other stroke subtypes (Choi et al., 2023). Notably, NVAF predominantly observed in aging populations, constitutes approximately 85% of global AF cases and NVAF-associated AIS accounts for over 50% of total stroke incidents (Lippi et al., 2021; Escudero-Martínez et al., 2023).

The pathophysiology underlying the increased stroke risk in AF is multifaceted. The irregular and chaotic atrial contraction characteristic of AF leads to blood stasis within the atrial chambers, particularly in the left atrial appendage (LAA), creating an environment conducive to thrombus formation (Bisson et al., 2018). This hemodynamic disturbance, coupled with endothelial dysfunction and a hypercoagulable state, constitutes Virchow’s triad for thrombogenesis in AF patients (Spartera et al., 2023). Additionally, AF is associated with systemic inflammation and oxidative stress, further exacerbating thrombotic risk through various molecular pathways including platelet activation, tissue factor expression, and disruption of the endothelial glycocalyx (Chousou et al., 2023).

While the CHA_2_DS_2_-VASc score remains the clinical benchmark for stratifying ischemic stroke risk in AF patients (Maheshwari et al., 2019), its predictive utility is constrained by a primary focus on clinical comorbidities, which have limited multi-dimensional predictive capacity. The score predominantly incorporates demographic factors and comorbid conditions-including congestive heart failure, hypertension, age, diabetes, prior stroke/TIA, vascular disease, and sex-without considering important physiological, anatomical, and hemodynamic parameters that may significantly influence stroke risk. Furthermore, the dichotomous nature of the scoring system fails to capture the gradation of risk associated with continuous variables such as left atrial size or biomarker levels.

Emerging evidence from recent studies demonstrates that ischemic stroke pathogenesis in NVAF patients involves multifactorial mechanisms spanning cardiac anatomical features, functional parameters, electrophysiological abnormalities, hemodynamic disturbances, and biomarker profiles (Wu et al., 2023). Left atrial enlargement and dysfunction have been significantly associated with thromboembolic events in AF patients, independent of traditional risk factors (Cho et al., 2021; Chung and Lee, 2022; Yu et al., 2025). Specific LAA morphologies and reduced LAA emptying velocities have demonstrated strong correlations with thrombus formation and subsequent embolic events (Tokunaga et al., 2020). Additionally, various biomarkers reflecting inflammation, coagulation activation, and myocardial injury have shown promise in enhancing stroke risk prediction models (Zhou et al., 2020). Among these, D-dimer—a marker of fibrin turnover and thrombus formation—has been validated as an independent predictor of ischemic stroke in patients with atrial fibrillation (Yuan et al., 2022; Shi et al., 2023). The white blood cell count, as a surrogate indicator of systemic inflammation, also correlates with atrial thrombogenesis by inducing endothelial dysfunction (Dolu et al., 2023; Li et al., 2022). Notably, clinical studies have demonstrated that the CHA_2_DS_2_-VASc/BS score, which incorporates B-type natriuretic peptide and cardiac troponin, yields improved predictive performance for stroke events in patients with non-valvular atrial fibrillation (Paulin et al., 2019). These findings further underscore the clinical value of integrating such multi-dimensional biomarkers into stroke risk prediction models.

Despite these advances in understanding the complex interplay of factors contributing to stroke risk in NVAF, current clinical risk stratification tools do not adequately integrate these multidimensional parameters. This gap in risk assessment potentially leads to suboptimal anticoagulation strategies in certain patient subgroups, highlighting the need for more comprehensive and precise risk prediction models.

This study aims to develop a refined predictive model for AIS in NVAF patients by systematic integration of clinical features, laboratory parameters, ambulatory electrocardiogram (AECG) recordings, echocardiographic findings, and Cardiac Multislice Spiral CT (Cardiac MSCT) metrics. Through this multifaceted approach, we seek to identify novel predictive factors and create a more accurate risk stratification tool to guide individualized preventive strategies and improve outcomes in this high-risk population.

## Materials and methods

### Study population

This single-center retrospective case-control study enrolled 415 patients with confirmed NVAF admitted to Northern Jiangsu People’s Hospital between October 2023 and October 2024. After applying inclusion and exclusion criteria, 374 NVAF patients were included in the final analysis, including 193 cases with AIS.

The inclusion criteria were as follows:

(1) Clinically confirmed AF by electrocardiography;
(2) Non-valvular etiology verified via echocardiography;
(3) AIS: occurring within 48 hours, diagnosed as cardiogenic stroke aligns with the relevant standards outlined in the “Chinese expert consensus on the diagnosis of cardiogenic stroke (2019)” (Liu et al., 2021) by neurologists. Imaging confirmation for AIS: All AIS patients included in the study had confirmatory neuroimaging findings: cerebral CT or MRI demonstrated acute ischemic lesions in vascular territories consistent with cardiogenic embolism (e.g., multiple cortical/subcortical infarcts in different vascular territories), and vascular imaging (CTA/MRA) showed no evidence of ≥ 50% stenosis in the corresponding cervical/cerebral arteries (ruling out large artery atherosclerosis). All patients having negative findings for hemorrhage on initial cerebral CT or MRI.

The exclusion criteria were as follows:

(1) Congenital heart disease, rheumatic heart disease, valvular heart disease, or post-procedural status (e.g., prosthetic valve replacement/repair);
(2) Recent acute coronary syndrome;
(3) History of catheter ablation for AF or percutaneous left atrial appendage (LAA) closure;
(4) Transient ischemic attack;
(5) Severe comorbidities (e.g., active infections, hepatic/renal insufficiency, malignancies, autoimmune/endocrine disorders);
(6) Recent major trauma or surgical intervention;
(7) Incomplete clinical data.

### Data extraction

(1) Clinical Characteristics: sex, age, body mass index (BMI), CHA_2_DS_2_-VASc score, admission SBP, and admission diastolic blood pressure (DBP).
(2) Medical History: history of hypertension, diabetes mellitus, coronary artery disease, heart failure, and ischemic stroke; AF type (paroxysmal or persistent); smoking and alcohol consumption status; use of oral antiplatelet or anticoagulant drugs.
(3) Laboratory Parameters: red blood cell count (RBC), hemoglobin (HB), white blood cell count (WBC), platelet count (PLT), mean platelet volume (MPV), aspartate aminotransferase (AST), alanine aminotransferase (ALT), creatinine (Cr), fasting blood glucose (GLU), triglycerides (TG), total cholesterol (TC), high-density lipoprotein (HDL), low-density lipoprotein (LDL), lipoprotein(a) (Lp(a)), N-terminal pro-B-type natriuretic peptide (NT-proBNP), troponin I (cTnI), D-dimer (DD) and urinary protein (PRO).
(4) Ambulatory Electrocardiogram (AECG): the presence of long RR intervals (≥ 2.0 s), minimum heart rate, maximum heart rate, and mean heart rate.
(5) Transthoracic Echocardiography: left atrial diameter (LAD), left ventricular end-diastolic diameter (LVDd), left ventricular end-systolic diameter (LVSd), left ventricular posterior wall thickness (LVPW), left ventricular ejection fraction (LVEF), and left ventricular fractional shortening (LVFS).
(6) Cardiac Multislice Spiral CT (Cardiac MSCT): LAA morphology (chicken wing vs. non-chicken wing), the presence of LAA filling defects, LAA orifice dimensions: length, width, and depth.

### Statistical analysis

Data analysis was performed using IBM SPSS Statistics 26.0 and R 4.4.2, with statistical significance set at *P* < 0.05. Normally distributed continuous variables were expressed as mean ± standard deviation (SD) and compared using independent t-tests; non-normally distributed variables were reported as median (IQR) and analyzed with Mann-Whitney U tests. Categorical variables were reported as frequencies (%) and compared via χ*^2^* or Fisher’s exact tests.

All patients were randomly divided into the training and the testing group at a ratio of 7:3. Univariate logistic regression analysis was first conducted in the training group, with variables demonstrating *P* < 0.2 subsequently entered into a LASSO regression model to mitigate multicollinearity and optimize predictive performance. Variables retained by LASSO regression were then subjected to multivariate logistic regression analysis using a backward elimination approach (likelihood ratio method) to identify independent risk factors. To ensure clinical relevance and biological plausibility, the variables selected by LASSO regression were independently reviewed by a panel of three board-certified physicians (neurology and cardiology), each with over a decade of specialized experience. A blinded assessment protocol was implemented, wherein each expert evaluated the variables separately. Any interpretative discrepancies were subsequently resolved through a structured consensus discussion to reach a unanimous final conclusion. Subsequently, the screened variables were included in multivariate logistic regression analysis, and the Backward (LR) method was adopted to identify independent risk factors and construct a nomogram for the prediction model. In the data of the training and the testing group respectively, the receiver-operating characteristic (ROC) curve was applied, the area under the ROC curve (AUC) was calculated, and calibration curves and decision curves (DCA) (via bootstrap resampling 1,000 iterations) were plotted to evaluate the discrimination, calibration, and clinical applicability of the model. In the full dataset, the AUC and decision curves of the nomogram prediction model were compared against those of individual risk factors in the full dataset to further evaluate rationality. Finally, the nomogram prediction model and CHA_2_DS_2_-VASc score model were compared using the NRI, IDI, AUC, calibration curve, and decision curve.

## Results

### Comparison of baseline data

**FIGURE 1:**
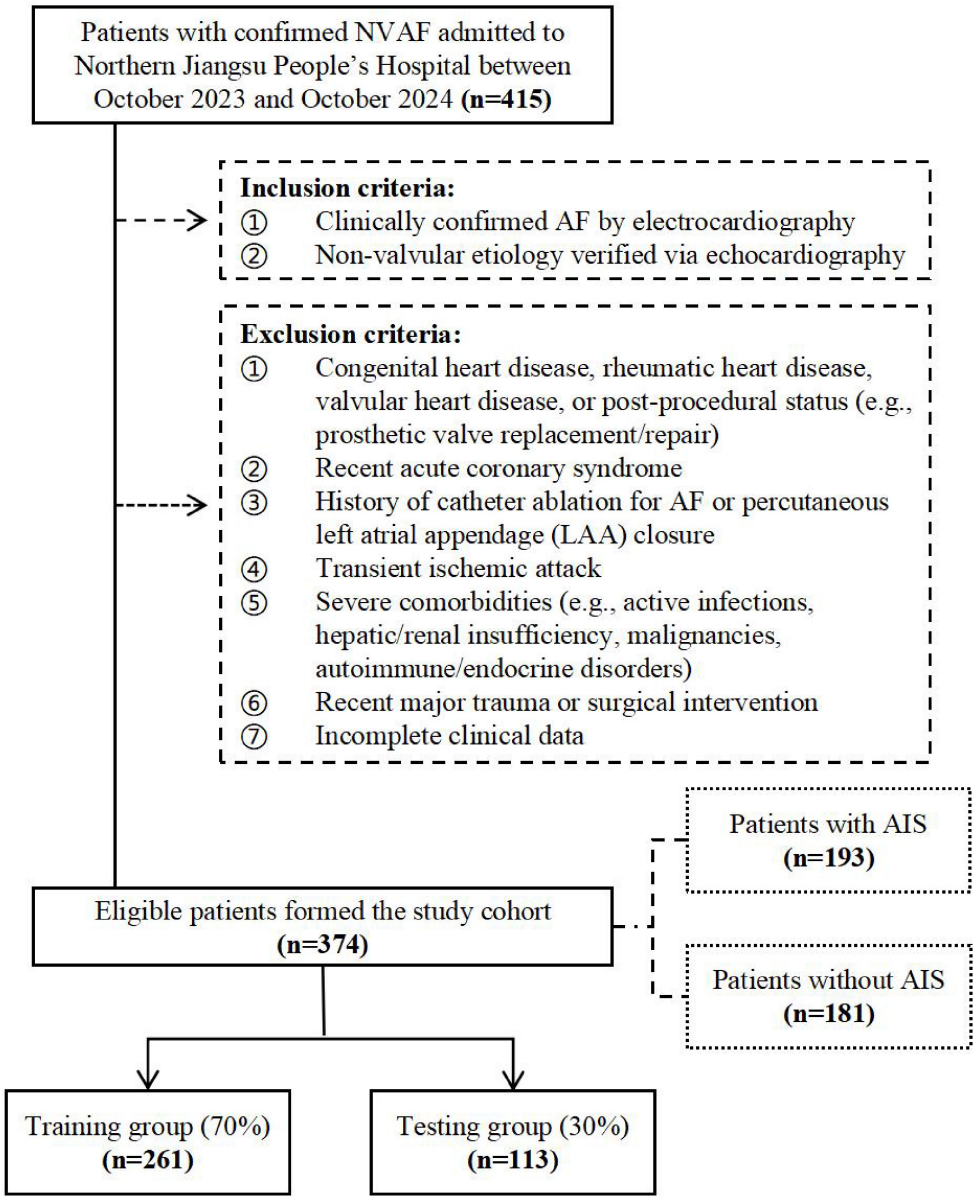
Flowchart with inclusion and exclusion criteria for the study. NVAF, non-valvular atrial fibrillation; AIS, acute ischemic stroke.

A total of 374 patients with NVAF were enrolled in this study, and 193 of them had AIS. All patients were randomly divided into the training group (*n* = 261) and the testing group (*n* = 113) at a ratio of 7:3 (Figure 1). Statistical analysis using SPSS Statistics (Version 26.0) revealed no significant differences (*p* > 0.05) between the training and the testing group (Table 1).

**TABLE 1:** Comparison of baseline data between the training group and the testing group.

Variables	Training group (*n* = 261)	Testing group (*n* = 113)	*P*-value
AIS, n%	134(51.3%)	59(52.2%)	0.966
Female sex, n%	115(44.1%)	48(42.5%)	0.865
Age, years	71.0(63.0; 77.0)	72.0(62.0; 76.0)	0.945
BMI, Kg/m^2^	23.9(22.0; 26.0)	24.8(22.5; 27.0)	0.065
History			
SBP, mmHg	138.0(125.0; 150.0)	137.0(122.0; 151.0)	0.751
DBP, mmHg	80.0(73.0; 89.0)	83.0(72.0; 91.0)	0.386
Hypertension, n%	173(66.3%)	81(71.7%)	0.365
Diabetes mellitus, n%	56(21.5%)	26(23.0%)	0.844
Stroke, n%	49(18.8%)	26(23.0%)	0.425
Heart failure, n%	42(16.1%)	14(12.4%)	0.445
Coronary heart disease, n%	68(26.1%)	28(24.8%)	0.896
Smoking, n%	63(24.1%)	30(26.5%)	0.715
Alcohol consumption, n%	44(16.9%)	25(22.1%)	0.289
Antiplatelet drugs, n%	27(10.3%)	14(12.4%)	0.688
Anticoagulant drugs, n%	67(25.7%)	26(23.0%)	0.677
AF type, n%			0.652
Persistent	128(49.0%)	59(52.2%)	
Paroxysmal	133(51.0%)	54(47.8%)	
Long RR interval, n%	93(35.6%)	45(39.8%)	0.513
Slowest heart rate, bpm	52.0(45.0; 58.0)	53.0(44.0; 58.0)	0.574
Average heart rate, bpm	72.0(65.0; 81.0)	73.0(65.0; 83.0)	0.516
fastest heart rate, bpm	126.0(104.0; 154.0)	131.0(105.0; 159.0)	0.485
LAD, mm	41.0(35.0; 43.0)	41.0(38.0; 44.0)	0.257
LVDd, mm	48.0(45.0; 51.0)	47.0(45.0; 49.0)	0.063
LVDs, mm	33.0(30.0; 35.0)	32.0(30.0; 34.0)	0.176
LVPW, mm	10.0(9.0; 10.0)	10.0(9.0; 10.0)	0.730
LVEF,%	60.0(57.0; 62.0)	60.0(56.0; 62.0)	0.683
LVFS,%	32.0(29.0; 33.0)	32.0(29.0; 33.0)	0.357
LAA morphology			0.852
Non-chicken wing	99(37.9%)	41(36.3%)	
Chicken wing	162(62.1%)	72(63.7%)	
LAA filling defect	49(18.8%)	19(16.8%)	0.760
LAA orifice length, mm	28.0(24.0; 32.1)	28.6(24.8; 33.7)	0.205
LAA orifice width, mm	21.0(17.0; 23.9)	21.4(18.6; 25.4)	0.059
LAA orifice depth, mm	30.3 ± 7.2	30.2 ± 6.6	0.851
RBC, 10^∧^12/L	4.4(4.0; 4.8)	4.5(4.1; 4.8)	0.163
HB, g/L	133.6 ± 18.3	136.9 ± 16.8	0.087
WBC,10^∧^9/L	6.2(5.0; 7.4)	6.2(5.0; 7.8)	0.385
PLT,10^∧^9/L	165.0(129.0; 211.0)	176.0(135.0; 214.0)	0.140
MVP, fL	11.2(10.1; 12.3)	10.9(10.1; 11.9)	0.335
ALT, U/L	19.3(14.0; 28.0)	22.5(16.0; 30.0)	0.055
AST, U/L	23.0(18.4; 29.0)	25.0(20.1; 31.0)	0.107
Cre, μmol/L	75.0(63.0; 88.0)	76.0(64.0; 89.0)	0.605
GLU, mmol/L	5.5(4.9; 6.7)	5.4(4.8; 6.5)	0.677
TG, mmol/L	1.3(0.9; 2.0)	1.2(0.9; 1.8)	0.234
TC, mmol/L	3.8(3.3; 4.6)	3.7(3.2; 4.4)	0.376
HDL-C, mmol/L	1.1(1.0; 1.4)	1.1(0.9; 1.4)	0.864
LDL-C, mmol/L	2.4(1.9; 2.8)	2.4(1.7; 2.9)	0.496
Lp(a), mmol/L	162.5(103.1; 289.9)	160.7(97.4; 258.6)	0.450
NT-proBNP, pg/mL	368.0(128.0; 998.0)	393.0(169.0; 1030.0)	0.598
Positive cTnI, n%	18(6.9%)	5(4.4%)	0.497
Positive PRO, n%	19(7.3%)	7(6.2%)	0.875
D-dimer, mg/L	0.3(0.2; 0.6)	0.3(0.2; 0.6)	0.953

### Univariate logistic regression analysis

In the training group, univariate logistic regression analysis was performed to identify potential risk factors for AIS in patients with NVAF. 24 independent variables with *P* < 0.2 were preliminarily screened, including age, BMI, admission SBP, admission DBP, history of stroke, smoking consumption, AF type, anticoagulant drugs, minimum heart rate, mean heart rate, LAD, LVPM, LVEF, LVFS, LAA morphology, LAA filling defect, LAA orifice length, LAA orifice width, WBC, AST, TC, LDL, NT-proBNP, and D-dimer (Table 2).

**TABLE 2:** Univariate logistic regression analysis.

Variables	*OR* (95%CI)	*P*-value
Sex	1.362(0.835–2.231)	0.217
Age	1.063(1.033–1.096)	< 0.001
BMI	0.946(0.881–1.013)	0.114
SBP	1.030(1.016–1.044)	< 0.001
DBP	1.017(0.998–1.036)	0.079
Hypertension	0.882(0.527–1.475)	0.634
Diabetes mellitus	1.228(0.679–2.237)	0.498
Stroke	2.854(1.480–5.769)	0.002
Heart failure	1.484(0.764–2.944)	0.249
Coronary heart disease	0.732(0.419–1.273)	0.271
Smoking	1.615(0.912–2.900)	0.103
Alcohol consumption	1.304(0.681–2.531)	0.426
Antiplatelet drugs	1.700(0.759–3.999)	0.206
Anticoagulant drugs	0.327(0.179–0.584)	<0.001
AFtype	0.384(0.232–0.631)	<0.001
Long RR interval	1.244(0.749–2.073)	0.401
Slowest heart rate	1.023(0.998–1.050)	0.071
Average heart rate	1.012(0.996–1.030)	0.143
Fastest heart rate	0.996(0.989–1.004)	0.316
LAD	1.114(1.067–1.168)	< 0.001
LVDd	0.996(0.938–1.057)	0.890
LVDs	1.037(0.975–1.104)	0.254
LVPW	0.748(0.557–0.996)	0.050
LVEF	0.957(0.913–1.000)	0.055
LVFS	0.935(0.871–1.000)	0.057
LAA morphology	0.625(0.375–1.033)	0.068
LAA filling defect	5.579(2.686–12.79)	< 0.001
LAA orifice length	1.068(1.024–1.115)	0.003
LAA orifice width	1.095(1.042–1.155)	0.001
LAA orifice depth	1.009(0.975–1.044)	0.614
RBC	0.885(0.585–1.333)	0.559
HB	0.992(0.978–1.005)	0.220
WBC	1.253(1.092–1.451)	0.002
PLT	1.002(0.998–1.007)	0.242
MVP	0.987(0.847–1.151)	0.869
ALT	1.009(0.992–1.028)	0.302
AST	1.031(1.008–1.057)	0.011
Cre	1.005(0.996–1.015)	0.298
GLU	1.033(0.935–1.147)	0.529
TG	1.110(0.869–1.432)	0.408
TC	0.728(0.543–0.966)	0.030
HDL-C	0.665(0.320–1.316)	0.254
LDL-C	0.771(0.561–1.052)	0.103
Lp(a)	1.000(0.998–1.001)	0.463
NT-proBNP	1.000(1.000–1.001)	0.042
Positive cTnI	1.200(0.458–3.241)	0.711
Positive PRO	1.686(0.655–4.667)	0.289
D-dimer	4.362(2.291–9.366)	<0.001

### LASSO regression analysis

**FIGURE 2:**
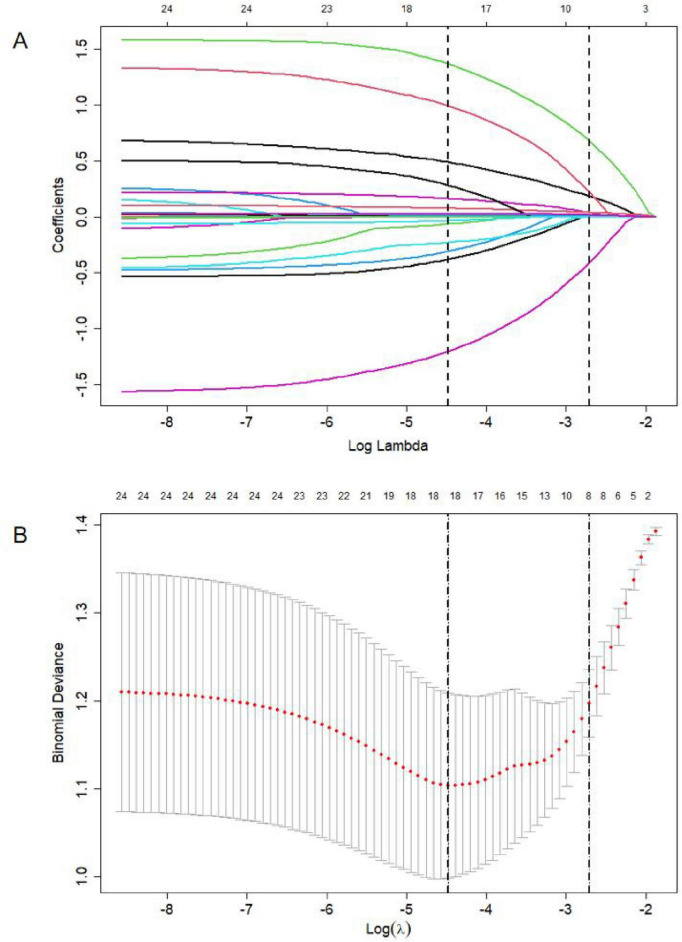
LASSO regression analysis. **(A)** LASSO coefficients produced by the regression analysis. **(B)** The optimal model was selected when the lambda value was equal to lambda.1Se, and 8 independent variables were included.

After integrating the 24 independent variables with *P* < 0.2 from the univariate logistic regression analysis into the LASSO regression. To ensure robustness, we centralized and normalized variables using 10-fold cross-validation. The outcomes of the LASSO regression indicated that age, admission SBP, history of stroke, anticoagulant drugs, LAD, LAA filling defect, WBC, and D-dimer were predictive variables for AIS in patients with NVAF (Figure 2).

### Multifactor logistic regression analysis

**FIGURE 3:**
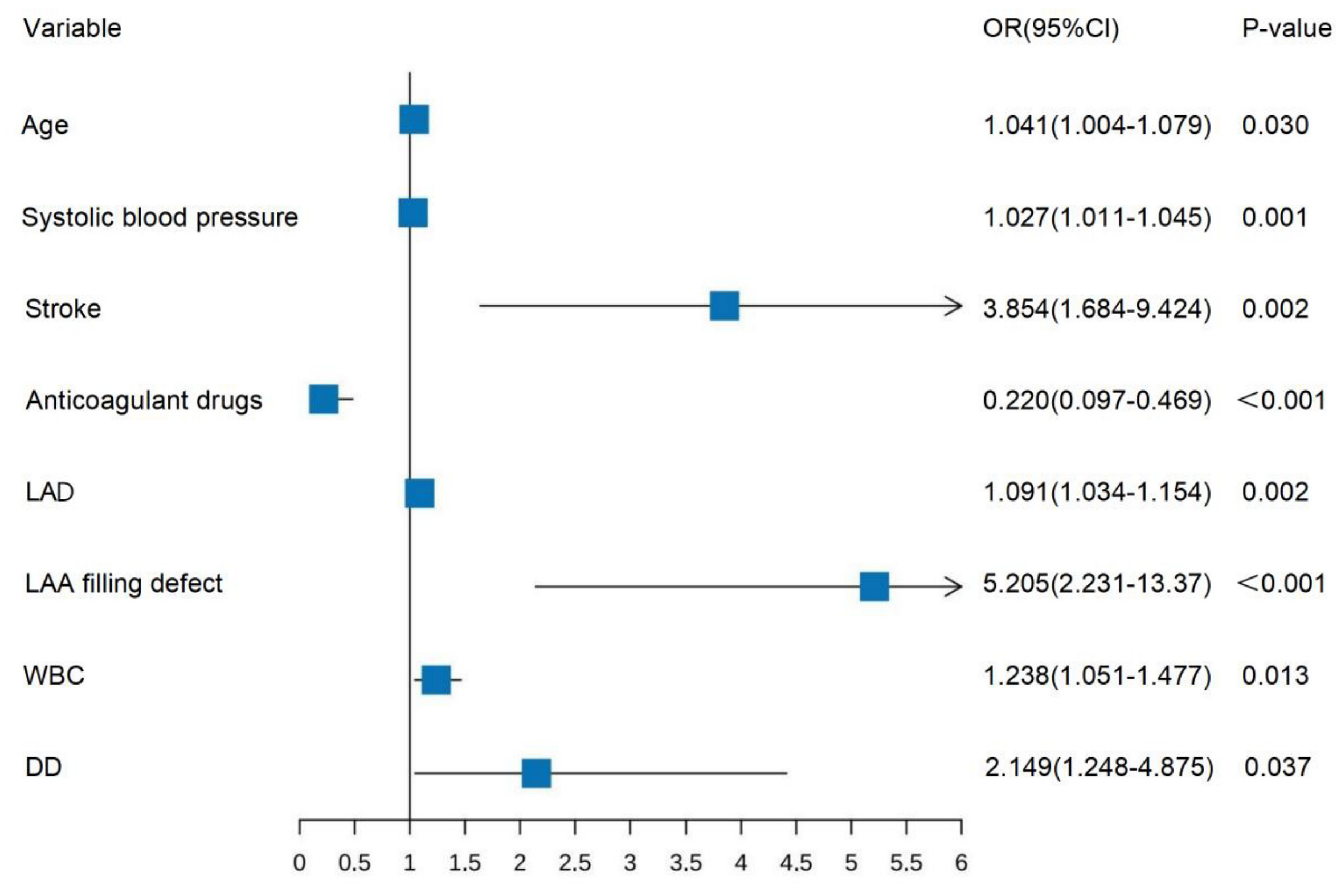
Forest plot of multifactor logistic regression analysis.

The 8 predictive variables identified through LASSO regression were incorporated into a multivariable logistic regression. The results revealed that age, admission SBP, history of stroke, anticoagulant drugs, LAD, LAA filling defect, WBC, and D-dimer were independent predictors for AIS in patients with NVAF (*P* < 0.05) (Figure 3).

### Construction of the new nomogram prediction model

**FIGURE 4:**
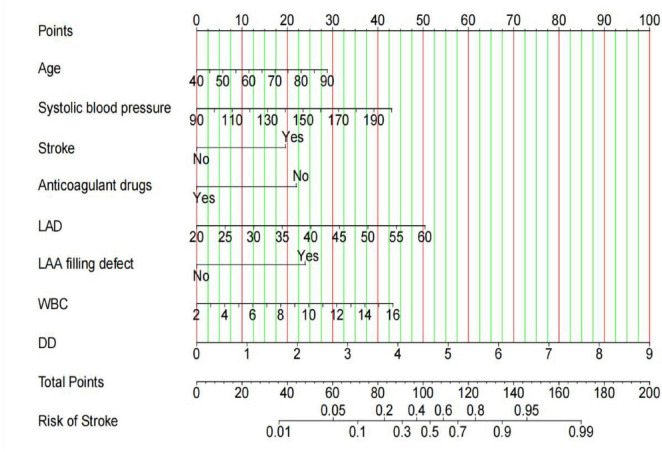
Nomogram for estimating the risk of ischemic stroke.

Using the independent predictors identified in the multivariable logistic regression analysis, a nomogram was generated to predict AIS in patients with NVAF (Figure 4).

Application of the nomogram prediction model: first, vertical projections of individual variable values onto the upper point scale yield corresponding scores for the eight independent predictors. Subsequently, the summation of these individual scores generates a total score. Finally, the vertical projection of the total score onto the probability axis at the model base provides the predicted probability of AIS in patients with NVAF. For example: a 65-year-old patient with NVAF, presenting with a history of stroke, no anticoagulant therapy, admission SBP of 130 mmHg, LAD of 40 mm (Echocardiography), absence of LAA filling defect (Cardiac MSCT), WBC count of 7.0 × 10∧9/L, and D-dimer level of 1.0 mg/L would achieve a total prediction score of 123 points, corresponding to an approximately 80% probability of ischemic stroke risk.

### Validation of the new nomogram prediction model

**FIGURE 5:**
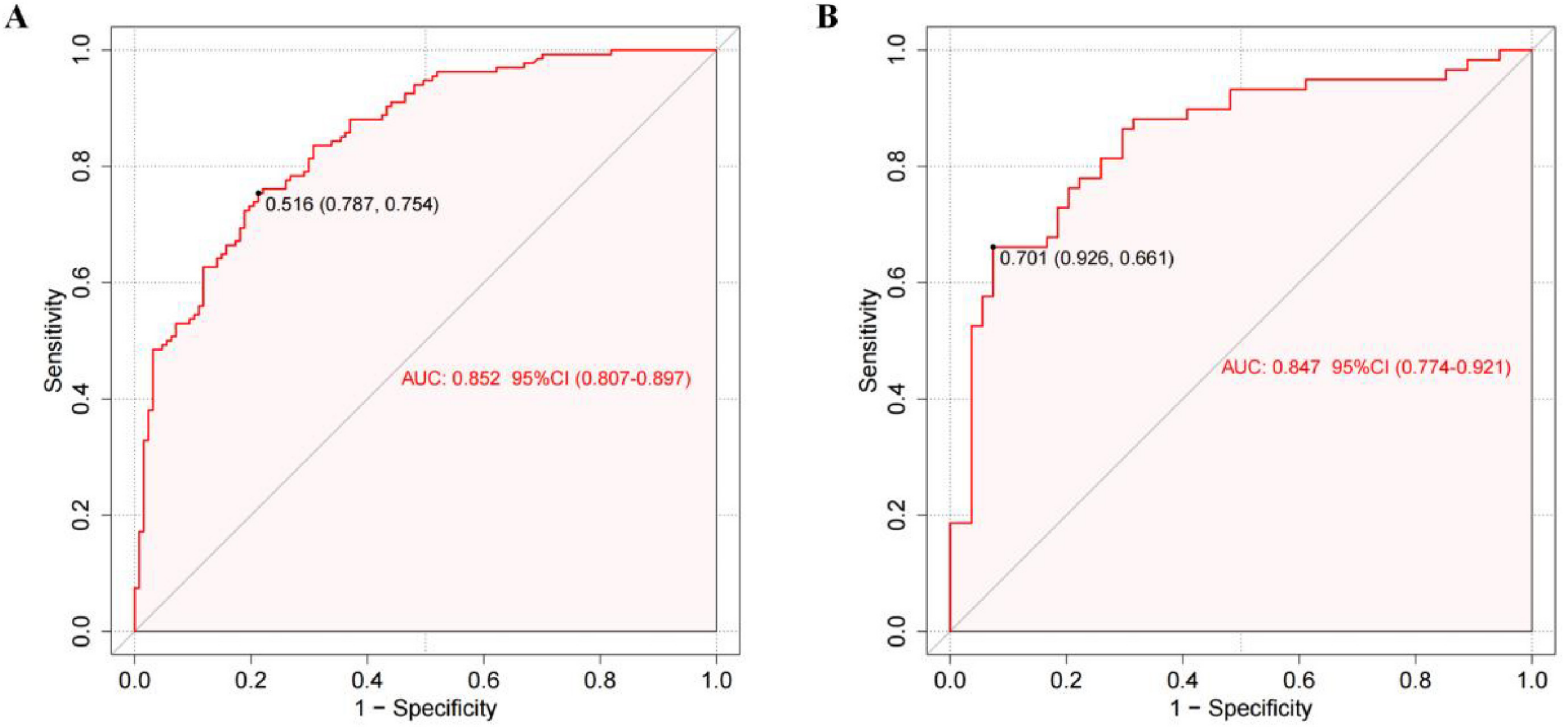
ROC curves of the nomogram prediction model for the training group **(A)** and testing group **(B)**.

**FIGURE 6:**
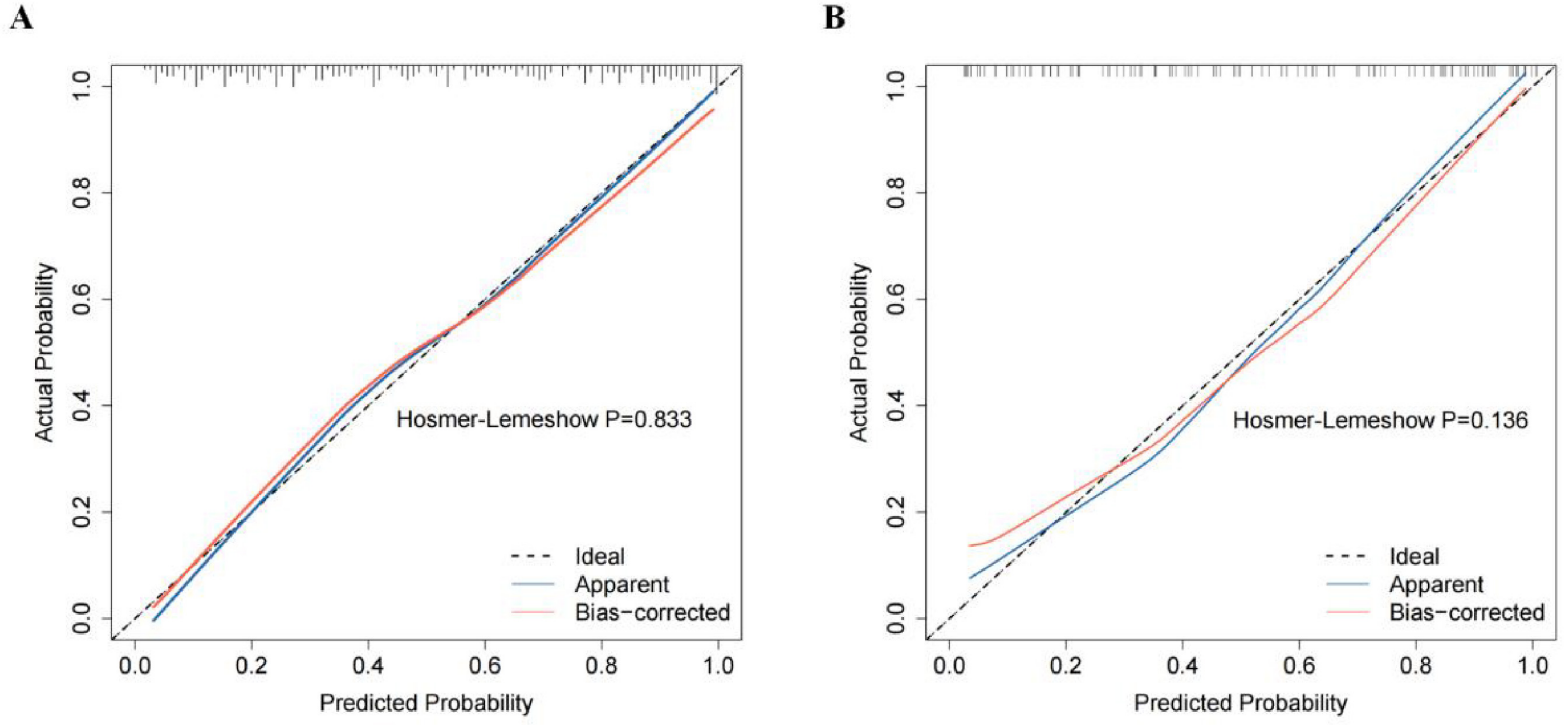
Calibration curves of the nomogram prediction model for the training group **(A)** and testing group **(B)**.

**FIGURE 7:**
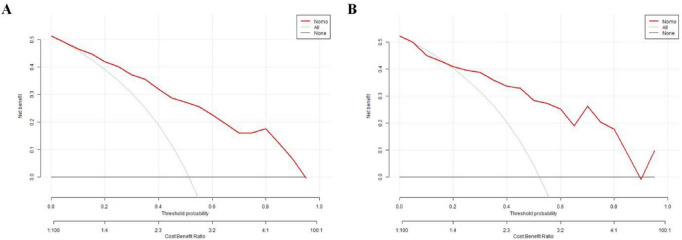
DCA of the nomogram prediction model for the training group **(A)** and testing group **(B)**.

The training group demonstrated an AUC of 0.852 [95% confidence interval (CI): 0.8067–0.8966], while the testing group achieved an AUC of 0.847 (95% CI: 0.7736–0.9207) on ROC analysis, indicating good discriminatory performance of the prediction model (Figure 5). Calibration curves were constructed using the bootstrap method with 1,000 resamples to assess goodness-of-fit. The calibration analysis revealed a close alignment between predicted and observed probabilities, with a calibration slope approximating unity. Hosemer-Lemeshow: Training group χ*^2^* = 4.257, *P* = 0.833; Testing group χ*^2^* = 12.350, *P* = 0.136. No statistically significant deviations between predicted and actual outcomes were observed in either cohort (*P* > 0.05), confirming satisfactory calibration (Figure 6). DCA was performed for both groups, with threshold probability plotted on the X-axis and net benefit rate on the Y-axis. The nomogram demonstrated higher net benefit rates across clinically relevant threshold probabilities compared to alternative strategies, supporting its robust clinical applicability for risk stratification of NVAF-related AIS (Figure 7).

**FIGURE 8:**
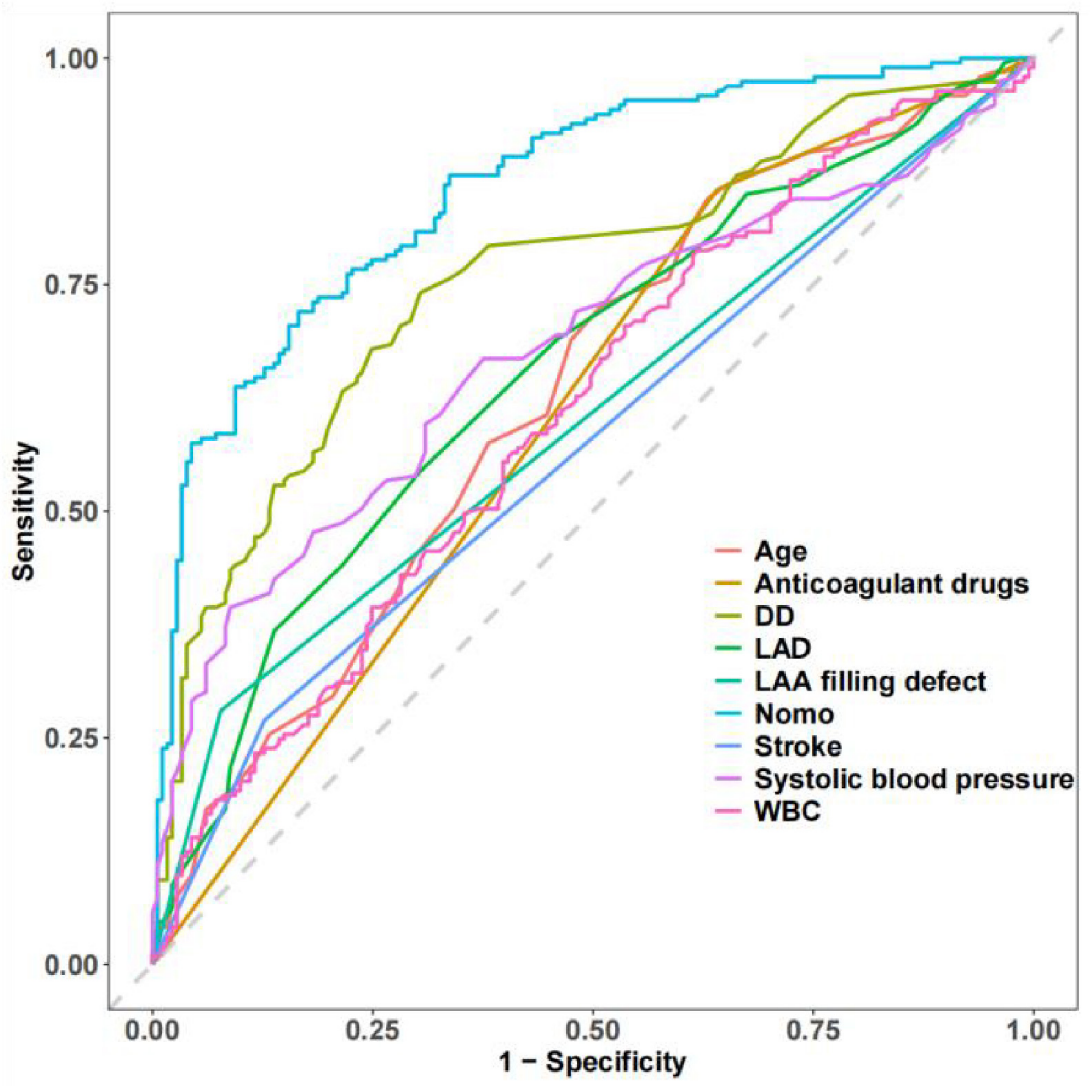
The nomogram prediction model achieved a higher AUC value compared to each individual predictor incorporated in the model.

**FIGURE 9:**
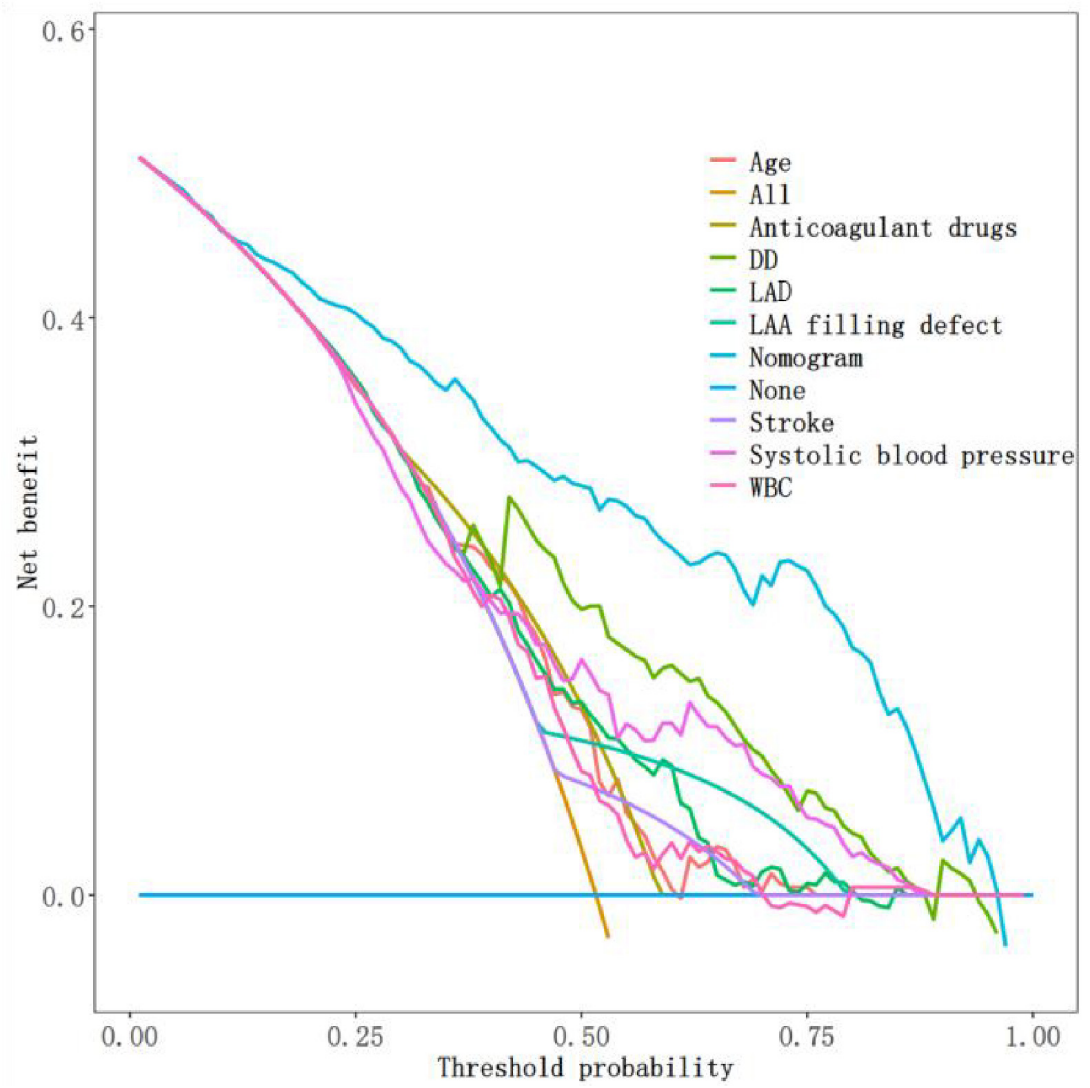
DCA of the nomogram prediction model and each individual predictor incorporated in the model.

ROC analysis demonstrated that the nomogram achieved a higher AUC value compared to each individual predictor incorporated in the model (Figure 8). DCA revealed superior net benefit for the nomogram across a broad threshold probability range when contrasted with risk stratification using each individual predictor (Figure 9). These findings further validate the rationality of the new nomogram prediction model.

### Comparison of the new nomogram prediction model and CHA_2_DS_2_-VASc score model

**FIGURE 10:**
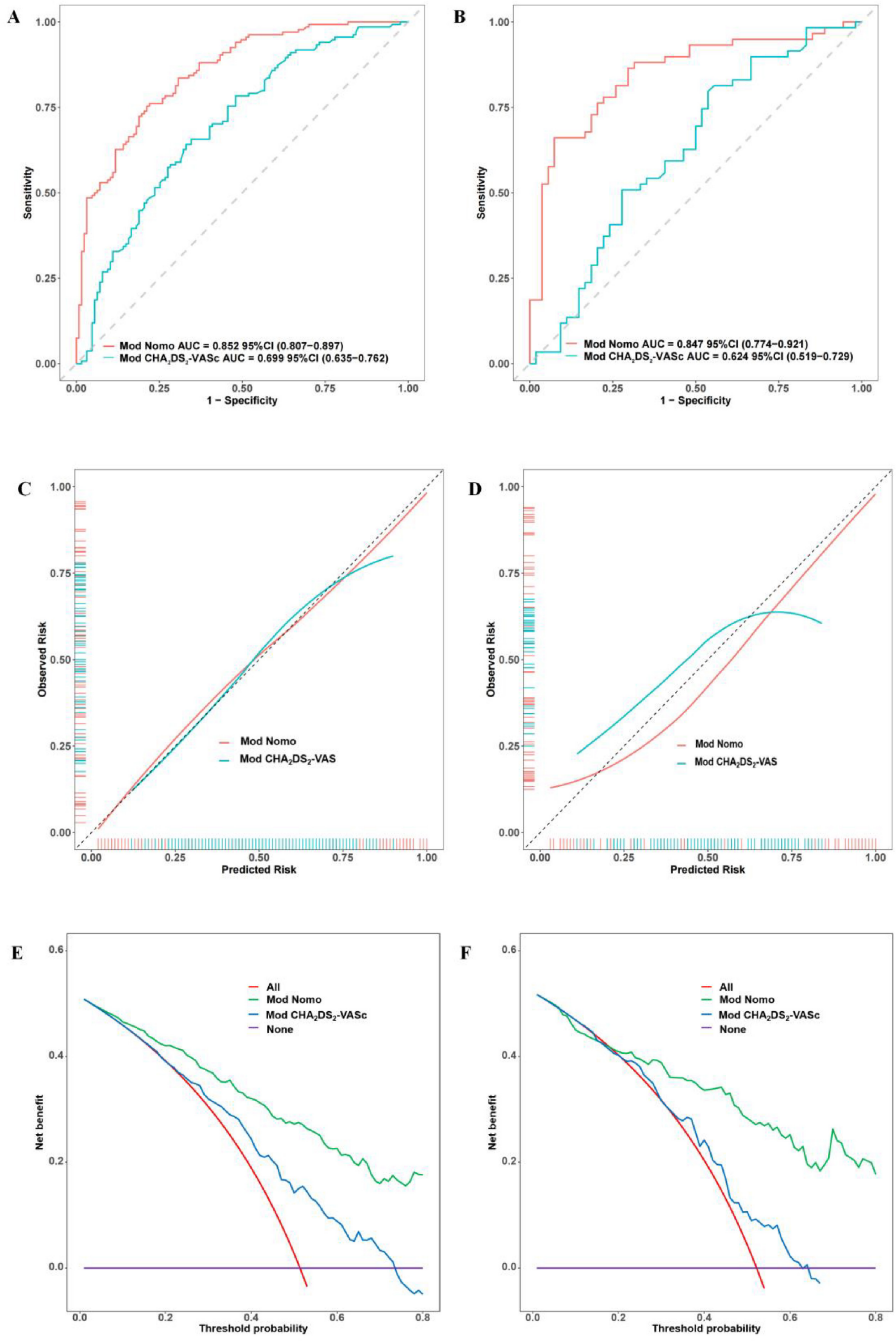
Comparison of the nomogram prediction and CHA_2_DS_2_-VASc Score model. Comparison of ROC curves between two models in the training group **(A)** and testing group **(B)**. Comparison of calibration curves between two models in the training group **(C)** and testing group **(D)**. Comparison of DCA between two models in the training group **(E)** and testing group **(F)**.

In this study, we compared the nomogram prediction model with the CHA_2_DS_2_-VASc scoring model in both the training and the testing group. In the training group, NRI: 0.262 (95% CI: 0.127–0.397, *P* < 0.001), IDI: 0.258 (95% CI: 0.200–0.315, *P* < 0.001), AUC of nomogram prediction model: 0.852 (95% CI: 0.807–0.897, AUC of CHA_2_DS_2_-VASc score model: 0.699 (95% CI: 0.635–0.762). In the testing group, NRI: 0.427 (95% CI: 0.216–0.638, *P* < 0.001), IDI: 0.351 (95% CI: 0.261–0.441, *P* < 0.001), AUC of nomogram prediction model: 0.847 (95% CI: 0.774–0.921), AUC of CHA_2_DS_2_-VASc score model: 0.624 (95% CI: 0.519–0.729) (Figure 10). These results showed that the nomogram prediction model demonstrated superior discrimination and calibration compared to the CHA_2_DS_2_-VASc Score model. Additionally, the nomogram prediction model showed enhanced clinical applicability in both the training and testing groups. Overall, the prediction ability of the nomogram prediction model was superior to that of the CHA_2_DS_2_-VASc score model.

## Discussion

Acute ischemic stroke (AIS) associated with atrial fibrillation (AF) is characterized by significantly higher rates of disability and mortality compared to other stroke subtypes (Elsheikh et al., 2024). This underscores the critical importance of early risk prediction and timely intervention to mitigate adverse outcomes in this high-risk population. The CHA_2_DS_2_-VASc score has been widely implemented in clinical practice for stratifying stroke risk in AF patients; however, its reliance on primarily categorical clinical variables presents substantial limitations. The score’s unidimensional approach fails to capture the complex pathophysiological mechanisms underlying thromboembolism in AF, resulting in a risk assessment tool that may lack precision for individualized clinical decision-making.

Contemporary research has elucidated that the pathophysiology of thrombus formation in AF extends beyond the traditional Virchow’s triad. While blood stasis from ineffective atrial contraction remains a fundamental mechanism, structural cardiac remodeling, endothelial dysfunction, systemic inflammation, and hypercoagulability all contribute significantly to thrombotic risk. This multifaceted pathophysiology necessitates a more comprehensive approach to risk assessment that integrates anatomical, functional, inflammatory, and hemodynamic parameters beyond what conventional clinical variables can provide.

In this study, we employed a systematic, data-driven approach to develop a more refined risk prediction tool for AIS in NVAF patients. Through sequential application of univariate logistic regression, LASSO regression for feature selection, and multivariate logistic regression, we identified eight independent predictors of AIS: age, admission systolic blood pressure (SBP), history of stroke, anticoagulant therapy status, left atrial diameter (LAD), left atrial appendage (LAA) filling defect, white blood cell count (WBC), and D-dimer levels. These parameters, which span clinical, anatomical, hemodynamic, and biochemical domains, were incorporated into a nomogram model that demonstrated excellent discrimination, calibration, and clinical utility upon internal validation.

Among the identified predictors, age and history of stroke are well-established risk factors that are also components of the CHA_2_DS_2_-VASc score. The heightened risk associated with prior stroke is consistently supported by robust evidence, including findings from the GARFIELD-AF registry reported by Hacke et al. (2020), which demonstrated a markedly increased risk of recurrent stroke in AF patients with prior stroke history (HR: 2.17, 95% CI: 1.80–2.63). Similarly, a nationwide Danish cohort study revealed that AF patients with previous stroke remained at substantial risk for recurrence despite secondary prevention strategies, with doubled risk among those who discontinued anticoagulation compared to those who maintained therapy (Hindsholm et al., 2024). The significant weight assigned to stroke history in the CHA_2_DS_2_-VASc score appropriately reflects its profound impact on risk stratification.

An intriguing finding from our analysis was the identification of admission SBP as a significant predictor of AIS in NVAF patients, while a history of hypertension did not demonstrate significant association. Although the acute-phase elevation of blood pressure during stroke is well-documented, our findings suggest that real-time blood pressure measurement may be more indicative of stroke risk than historical diagnosis of hypertension. This observation aligns with research by Ishii et al. (2017), emphasizing the critical importance of effective blood pressure management in AF patients. The relationship between blood pressure variability and stroke risk in this population warrants further investigation, as emerging evidence suggests that blood pressure fluctuations may independently contribute to adverse cardiovascular outcomes beyond mean pressure values.

The incorporation of LAA filling defects as detected by Cardiac MSCT into our prediction model represents an important advancement in risk stratification. Previous research has established that approximately 90% of cardioembolic events in NVAF patients originate from thrombi in the LAA (Zhang et al., 2024). While Cardiac MSCT offers a straightforward, time-efficient, and non-invasive method for detecting LAA thrombosis (Hajhosseiny et al., 2024), the potential for false-positive diagnoses must be acknowledged. Nicol et al. (2024) demonstrated that even a false-positive LAA thrombus finding remained independently associated with cardiogenic stroke (OR: 3.33, 95% CI: 1.42–7.81, *P* = 0.006). By incorporating LAA filling defects into our model, we capture important anatomical information that reflects thrombotic potential not adequately represented by clinical variables alone.

Left atrial enlargement emerged as another significant predictor in our model. Beyond being a consequence of AF, left atrial dilation serves as an independent predictor of thrombotic events (Tan et al., 2023). The left atrial diameter, readily assessed by echocardiography, has been shown to correlate with thrombotic risk in multiple studies. Research focusing on NVAF patients with low CHA_2_DS_2_-VASc scores found that increased left atrial diameter was independently associated with LAA thrombosis (OR: 1.088, 95% CI: 1.032–1.146, *P* < 0.05) (Kamili et al., 2024). Hirota et al. (2021) further demonstrated a dose-response relationship between left atrial dimension and ischemic stroke risk. Future refinements of prediction models might benefit from incorporating more nuanced assessments of left atrial structure and function, potentially including fibrosis evaluation through advanced imaging techniques.

The identification of WBC count and D-dimer levels as independent predictors highlights the crucial role of inflammatory and coagulation biomarkers in thrombotic risk assessment. Inflammation compromises vascular endothelial integrity and promotes hypercoagulability, creating favorable conditions for thrombus formation (Kelly et al., 2021). Dolu et al. (2023) reported elevated WBC count as an independent risk factor for left atrial thrombosis in NVAF (OR: 1.26, 95% CI: 1.05–1.51), and Li et al. (2022) demonstrated a positive correlation between leukocyte count and AIS risk. Similarly, D-dimer has been consistently associated with thromboembolic risk in AF patients (Yuan et al., 2022; Shi et al., 2023), maintaining its predictive value even in patients receiving anticoagulation therapy (Christersson et al., 2014; Siegbahn et al., 2016). The integration of these biomarkers into risk assessment represents a significant advancement toward personalized stroke prevention strategies in AF. Serial D-dimer measurements, in particular, could potentially enable dynamic risk assessment, offering advantages over static clinical risk scores.

Our study confirmed that absence of anticoagulant therapy is an independent risk factor for AIS in NVAF patients, consistent with established guidelines (Wang et al., 2024; Van Gelder et al., 2024). Concerningly, our data revealed that less than 25% of NVAF patients received appropriate anticoagulation, with many inappropriately receiving antiplatelet therapy instead. While anticoagulation decisions must be individualized, the widespread underutilization of guideline-directed therapy underscores the need for improved risk stratification and clinical decision support tools. Our nomogram model aims to address this gap by providing more precise risk assessment to guide appropriate preventive interventions, with its predictive framework built on a comprehensive importance ranking of 8 independent predictors derived from standardized regression coefficients of multivariate logistic regression and normalized LASSO feature importance scores; notably, LAA filling defect and D-dimer topped the ranking, followed by history of stroke, admission systolic blood pressure, age, LAD, WBC, with absence of anticoagulant therapy ranking the lowest.

The integration of multiple risk domains—clinical, anatomical, functional, and biochemical—into a single predictive nomogram represents a significant advancement in personalized stroke risk assessment for NVAF patients. The superior performance of our model compared to the CHA_2_DS_2_-VASc score has important clinical implications. While the CHA_2_DS_2_-VASc score remains valuable for its simplicity and widespread familiarity, our findings suggest that more comprehensive assessment tools incorporating hierarchically ranked risk factors (with left atrial appendage filling defect and D-dimer as the most impactful indicators) may better guide anticoagulation decisions, particularly in patients whose risk stratification remains ambiguous using traditional methods. Furthermore, the nomogram format provides an intuitive visual representation of risk that can facilitate shared decision-making between clinicians and patients.

Future research should focus on prospective validation of this model in diverse patient populations, integration of additional emerging biomarkers and imaging parameters, and development of dynamic risk assessment tools capable of capturing temporal changes in stroke risk. Machine learning approaches may further enhance predictive accuracy by identifying complex patterns and interactions among risk factors not readily apparent through conventional statistical methods. Additionally, exploration of genetic and epigenetic factors in AF-related stroke risk represents an exciting frontier for future risk stratification models.

This study has several limitations. First, as a single-center retrospective analysis, selection bias may influence our results. Second, the stability of the variable selection process (LASSO followed by logistic regression), while effective, may be sensitive to sample variations. Third, while the model demonstrated satisfactory performance in internal validation, it has not undergone external validation, limiting its generalizability to other clinical settings. Fourth, despite considering numerous potential factors, some indicators with possible predictive value, such as IL-6 (Singleton et al., 2021) and P-wave index (Maheshwari et al., 2019), were not included due to incomplete clinical data. Future large-scale, multicenter prospective studies are needed to refine and further validate the model, ultimately benefiting a broader patient population.

## Conclusion

Age, admission systolic blood pressure, history of stroke, anticoagulant therapy status, left atrial diameter, left atrial appendage filling defect, white blood cell count, and D-dimer levels were identified as independent predictors of acute ischemic stroke in patients with non-valvular atrial fibrillation. The novel nomogram prediction model incorporating these multidimensional parameters demonstrated superior predictive performance compared to the conventional CHA_2_DS_2_-VASc score model. As a complementary tool, this approach allows for the personalization of anticoagulation strategies, which may lead to improved stroke prevention.

## Data Availability

The original contributions presented in the study are included in the article/supplementary material, further inquiries can be directed to the corresponding author.
